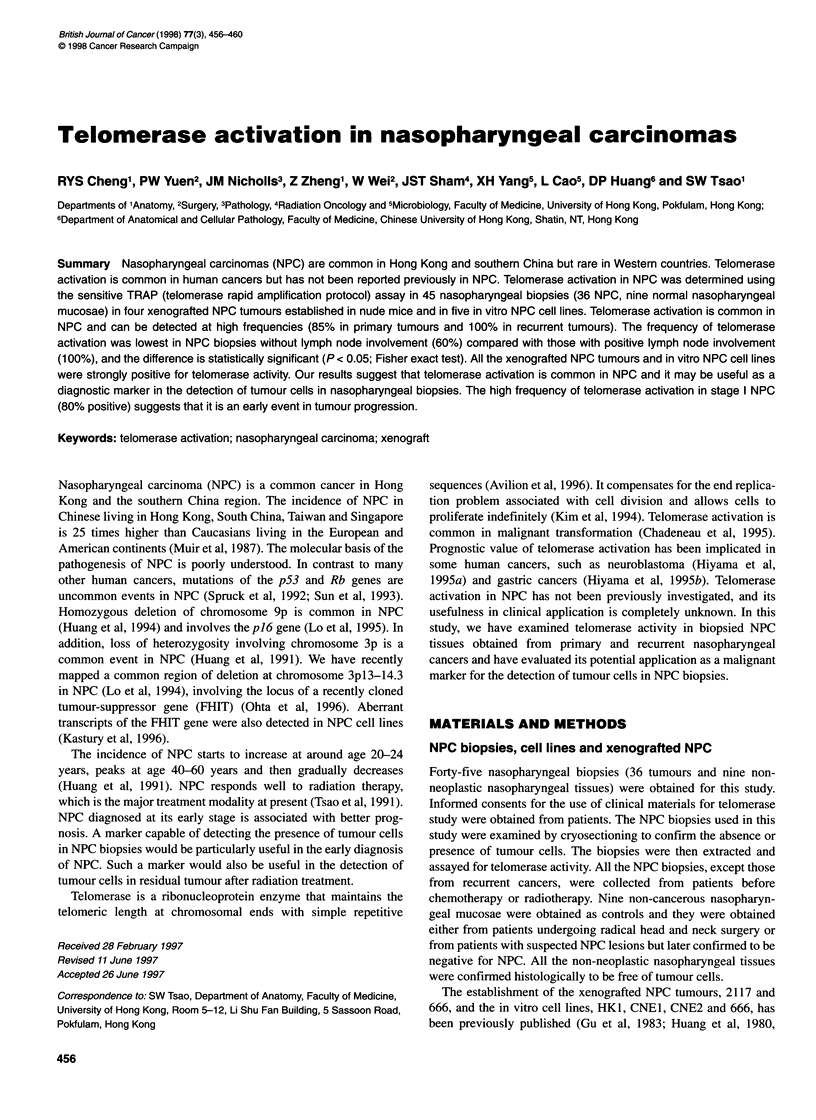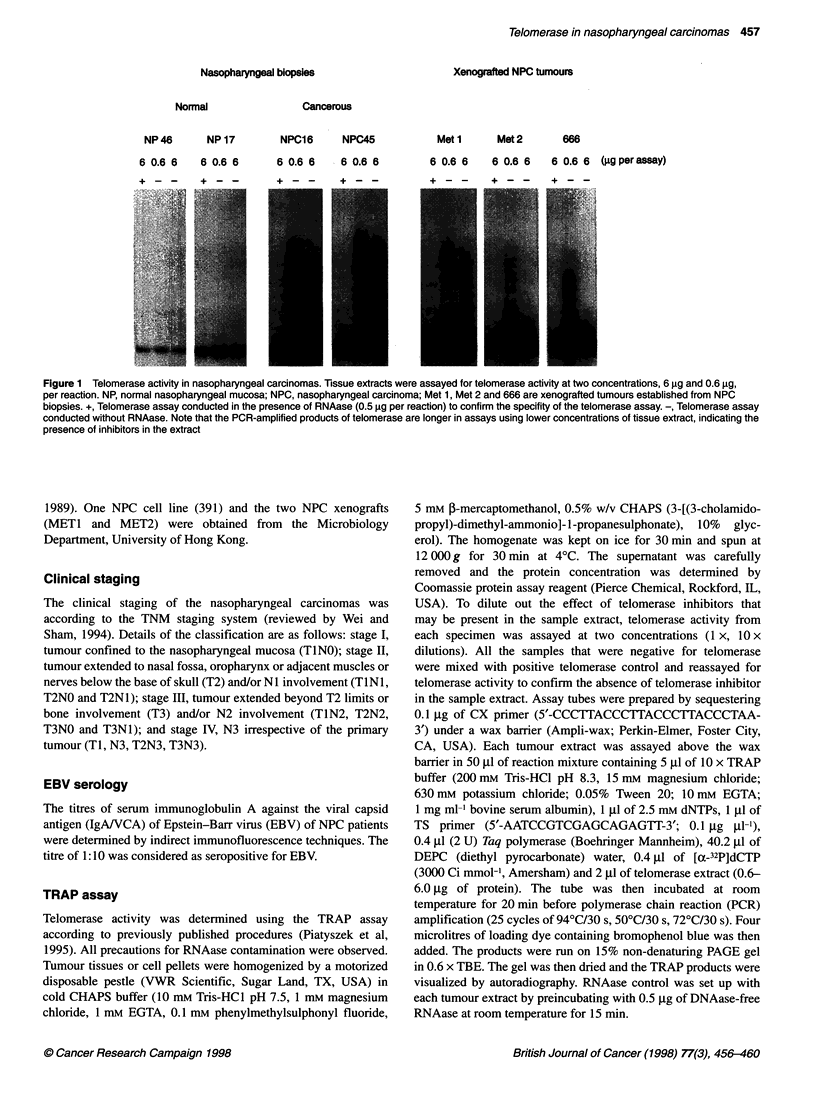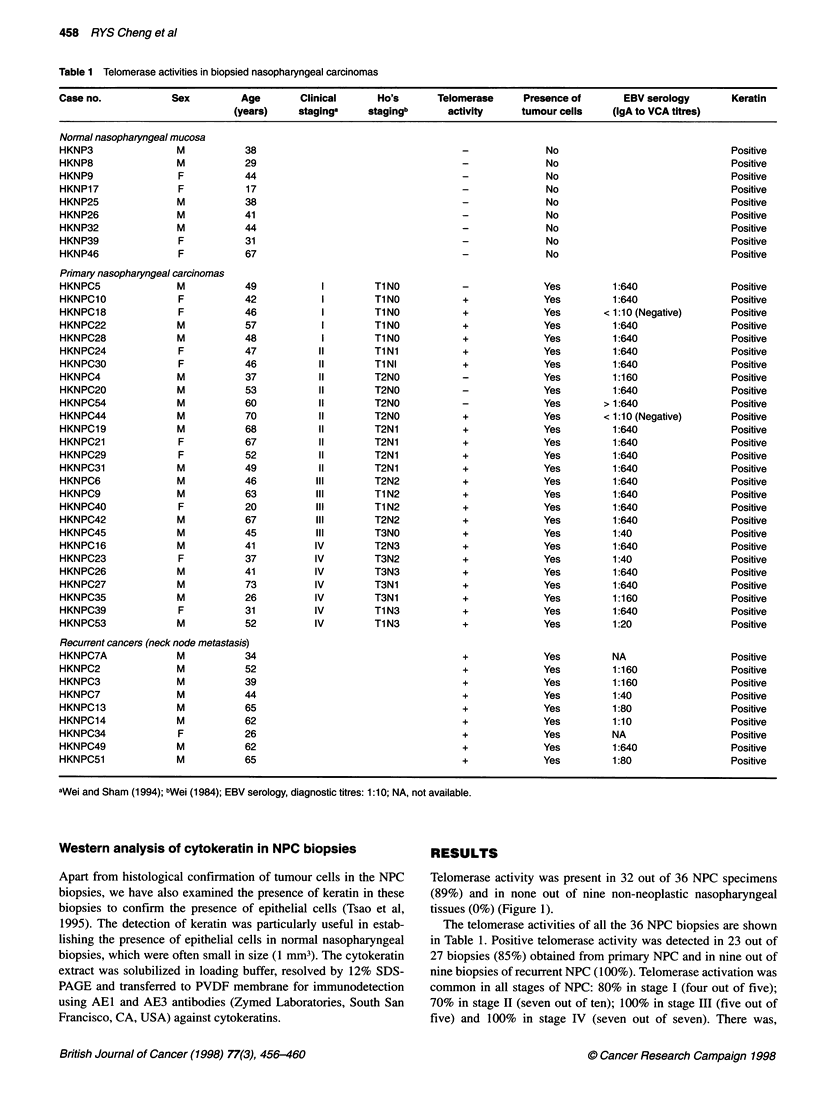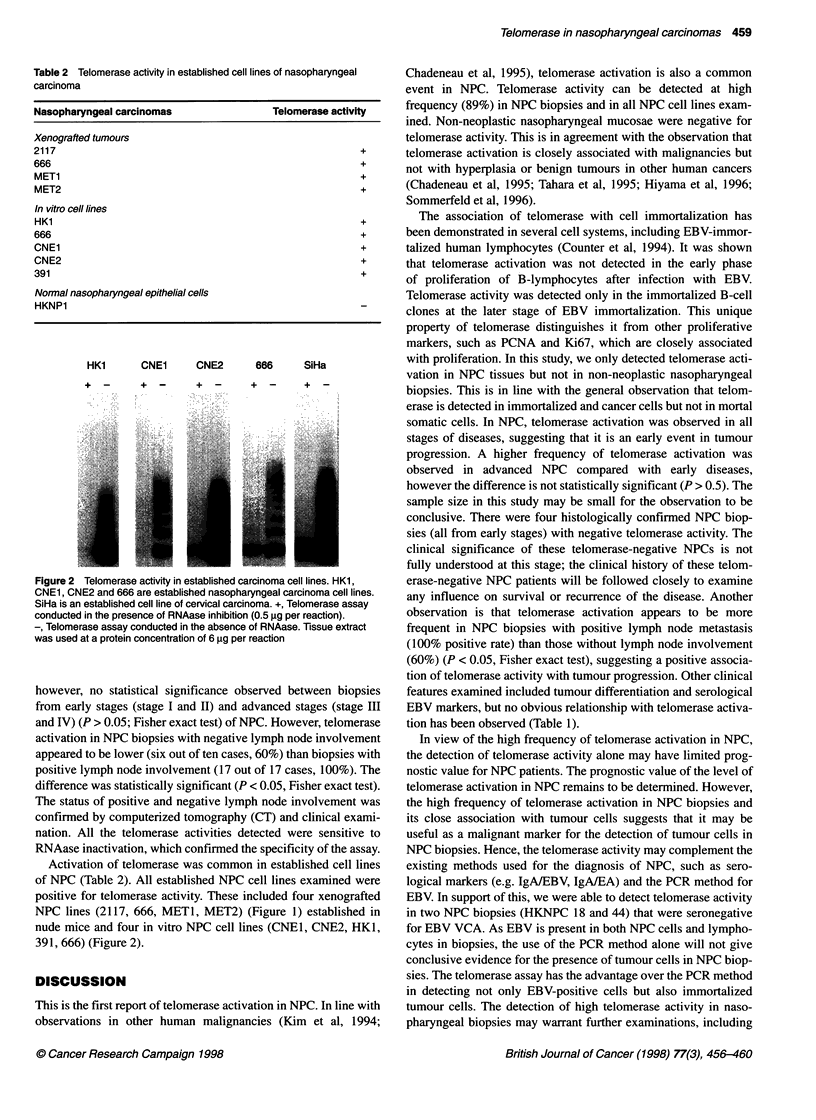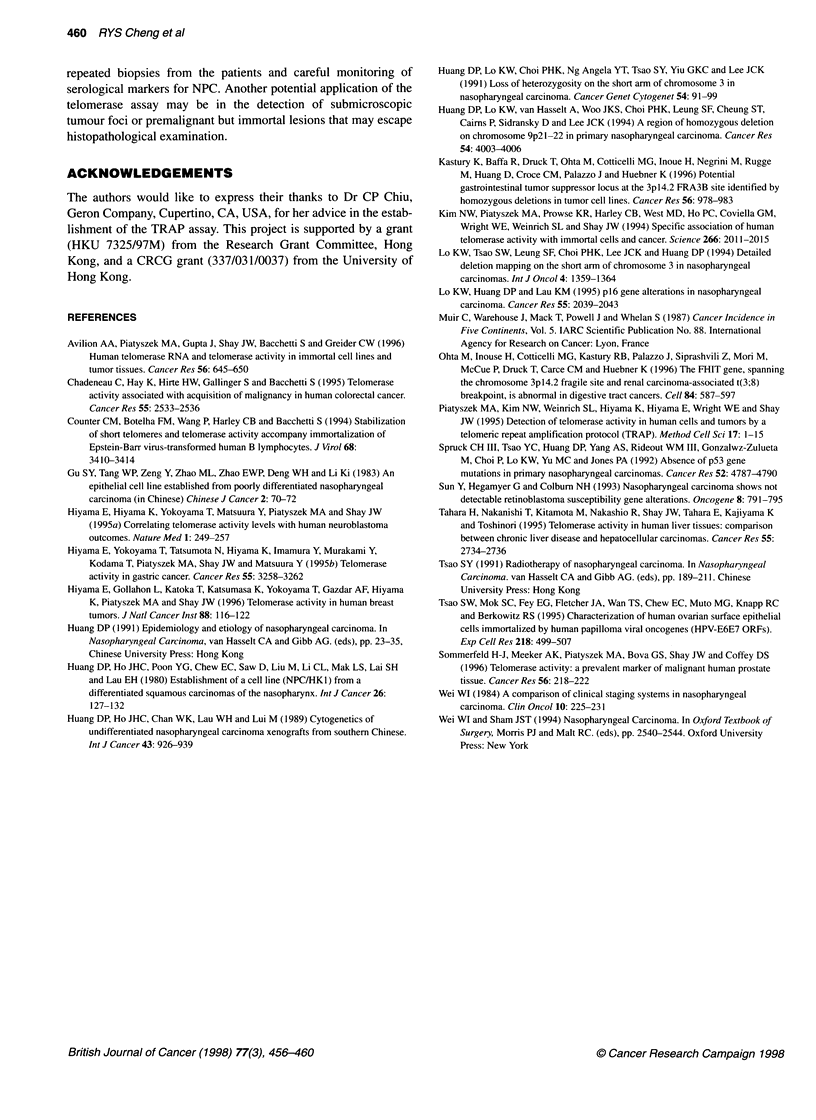# Telomerase activation in nasopharyngeal carcinomas.

**DOI:** 10.1038/bjc.1998.72

**Published:** 1998

**Authors:** R. Y. Cheng, P. W. Yuen, J. M. Nicholls, Z. Zheng, W. Wei, J. S. Sham, X. H. Yang, L. Cao, D. P. Huang, S. W. Tsao

**Affiliations:** Department of Anatomy, Faculty of Medicine, University of Hong Kong, Pokfulam.

## Abstract

**Images:**


British Journal of Cancer (1998) 77(3), 456–460
© 1998 Cancer Research Campaign

### Telomerase activation in nasopharyngeal carcinomas

**RYS Cheng[1], PW Yuen[2], JM Nicholls[3], Z Zheng[1], W Wei[2], JST Sham[4], XH Yang[5], L Cao[5], DP Huang[6] and SW Tsao[1]**

Departments of [1]Anatomy, [2]Surgery, [3]Pathology, [4]Radiation Oncology and [5]Microbiology, Faculty of Medicine, University of Hong Kong, Pokfulam, Hong Kong; [6]Department of Anatomical and Cellular Pathology, Faculty of Medicine, Chinese University of Hong Kong, Shatin, NT, Hong Kong

**Summary** Nasopharyngeal carcinomas (NPC) are common in Hong Kong and southern China but rare in Western countries. Telomerase activation is common in human cancers but has not been reported previously in NPC. Telomerase activation in NPC was determined using the sensitive TRAP (telomerase rapid amplification protocol) assay in 45 nasopharyngeal biopsies (36 NPC, nine normal nasopharyngeal mucosae) in four xenografted NPC tumours established in nude mice and in five in vitro NPC cell lines. Telomerase activation is common in NPC and can be detected at high frequencies (85% in primary tumours and 100% in recurrent tumours). The frequency of telomerase activation was lowest in NPC biopsies without lymph node involvement (60%) compared with those with positive lymph node involvement (100%), and the difference is statistically significant ($P < 0.05$; Fisher exact test). All the xenografted NPC tumours and in vitro NPC cell lines were strongly positive for telomerase activity. Our results suggest that telomerase activation is common in NPC and it may be useful as a diagnostic marker in the detection of tumour cells in nasopharyngeal biopsies. The high frequency of telomerase activation in stage I NPC (80% positive) suggests that it is an early event in tumour progression.

**Keywords:** telomerase activation; nasopharyngeal carcinoma; xenograft

Nasopharyngeal carcinoma (NPC) is a common cancer in Hong Kong and the southern China region. The incidence of NPC in Chinese living in Hong Kong, South China, Taiwan and Singapore is 25 times higher than Caucasians living in the European and American continents (Muir et al, 1987). The molecular basis of the pathogenesis of NPC is poorly understood. In contrast to many other human cancers, mutations of the *p53* and *Rb* genes are uncommon events in NPC (Spruck et al, 1992; Sun et al, 1993). Homozygous deletion of chromosome 9p is common in NPC (Huang et al, 1994) and involves the *p16* gene (Lo et al, 1995). In addition, loss of heterozygosity involving chromosome 3p is a common event in NPC (Huang et al, 1991). We have recently mapped a common region of deletion at chromosome 3p13–14.3 in NPC (Lo et al, 1994), involving the locus of a recently cloned tumour-suppressor gene (FHIT) (Ohta et al, 1996). Aberrant transcripts of the FHIT gene were also detected in NPC cell lines (Kastury et al, 1996).

The incidence of NPC starts to increase at around age 20–24 years, peaks at age 40–60 years and then gradually decreases (Huang et al, 1991). NPC responds well to radiation therapy, which is the major treatment modality at present (Tsao et al, 1991). NPC diagnosed at its early stage is associated with better prognosis. A marker capable of detecting the presence of tumour cells in NPC biopsies would be particularly useful in the early diagnosis of NPC. Such a marker would also be useful in the detection of tumour cells in residual tumour after radiation treatment.

Telomerase is a ribonucleoprotein enzyme that maintains the telomeric length at chromosomal ends with simple repetitive sequences (Avilion et al, 1996). It compensates for the end replication problem associated with cell division and allows cells to proliferate indefinitely (Kim et al, 1994). Telomerase activation is common in malignant transformation (Chadeneau et al, 1995). Prognostic value of telomerase activation has been implicated in some human cancers, such as neuroblastoma (Hiyama et al, 1995*a*) and gastric cancers (Hiyama et al, 1995*b*). Telomerase activation in NPC has not been previously investigated, and its usefulness in clinical application is completely unknown. In this study, we have examined telomerase activity in biopsied NPC tissues obtained from primary and recurrent nasopharyngeal cancers and have evaluated its potential application as a malignant marker for the detection of tumour cells in NPC biopsies.

*Received 28 February 1997*
*Revised 11 June 1997*
*Accepted 26 June 1997*

*Correspondence to:* SW Tsao, Department of Anatomy, Faculty of Medicine, University of Hong Kong, Room 5–12, Li Shu Fan Building, 5 Sassoon Road, Pokfulam, Hong Kong

### MATERIALS AND METHODS

### NPC biopsies, cell lines and xenografted NPC

Forty-five nasopharyngeal biopsies (36 tumours and nine non-neoplastic nasopharyngeal tissues) were obtained for this study. Informed consents for the use of clinical materials for telomerase study were obtained from patients. The NPC biopsies used in this study were examined by cryosectioning to confirm the absence or presence of tumour cells. The biopsies were then extracted and assayed for telomerase activity. All the NPC biopsies, except those from recurrent cancers, were collected from patients before chemotherapy or radiotherapy. Nine non-cancerous nasopharyngeal mucosae were obtained as controls and they were obtained either from patients undergoing radical head and neck surgery or from patients with suspected NPC lesions but later confirmed to be negative for NPC. All the non-neoplastic nasopharyngeal tissues were confirmed histologically to be free of tumour cells.

The establishment of the xenografted NPC tumours, 2117 and 666, and the in vitro cell lines, HK1, CNE1, CNE2 and 666, has been previously published (Gu et al, 1983; Huang et al, 1980,

**Figure 1** Telomerase activity in nasopharyngeal carcinomas. Tissue extracts were assayed for telomerase activity at two concentrations, 6 μg and 0.6 μg, per reaction. NP, normal nasopharyngeal mucosa; NPC, nasopharyngeal carcinoma; Met 1, Met 2 and 666 are xenografted tumours established from NPC biopsies. +, Telomerase assay conducted in the presence of RNAase (0.5 μg per reaction) to confirm the specifity of the telomerase assay. –, Telomerase assay conducted without RNAase. Note that the PCR-amplified products of telomerase are longer in assays using lower concentrations of tissue extract, indicating the presence of inhibitors in the extract

1989). One NPC cell line (391) and the two NPC xenografts (MET1 and MET2) were obtained from the Microbiology Department, University of Hong Kong.

### Clinical staging

The clinical staging of the nasopharyngeal carcinomas was according to the TNM staging system (reviewed by Wei and Sham, 1994). Details of the classification are as follows: stage I, tumour confined to the nasopharyngeal mucosa (T1N0); stage II, tumour extended to nasal fossa, oropharynx or adjacent muscles or nerves below the base of skull (T2) and/or N1 involvement (T1N1, T2N0 and T2N1); stage III, tumour extended beyond T2 limits or bone involvement (T3) and/or N2 involvement (T1N2, T2N2, T3N0 and T3N1); and stage IV, N3 irrespective of the primary tumour (T1, N3, T2N3, T3N3).

### EBV serology

The titres of serum immunoglobulin A against the viral capsid antigen (IgA/VCA) of Epstein–Barr virus (EBV) of NPC patients were determined by indirect immunofluorescence techniques. The titre of 1:10 was considered as seropositive for EBV.

### TRAP assay

Telomerase activity was determined using the TRAP assay according to previously published procedures (Piatyszek et al, 1995). All precautions for RNAase contamination were observed. Tumour tissues or cell pellets were homogenized by a motorized disposable pestle (VWR Scientific, Sugar Land, TX, USA) in cold CHAPS buffer (10 mM Tris-HC1 pH 7.5, 1 mM magnesium chloride, 1 mM EGTA, 0.1 mM phenylmethylsulphonyl fluoride, 5 mM β-mercaptomethanol, 0.5% w/v CHAPS (3-[(3-cholamidopropyl)-dimethyl-ammonio]-1-propanesulphonate), 10% glycerol). The homogenate was kept on ice for 30 min and spun at 12 000 *g* for 30 min at 4°C. The supernatant was carefully removed and the protein concentration was determined by Coomassie protein assay reagent (Pierce Chemical, Rockford, IL, USA). To dilute out the effect of telomerase inhibitors that may be present in the sample extract, telomerase activity from each specimen was assayed at two concentrations (1 ×, 10 × dilutions). All the samples that were negative for telomerase were mixed with positive telomerase control and reassayed for telomerase activity to confirm the absence of telomerase inhibitor in the sample extract. Assay tubes were prepared by sequestering 0.1 μg of CX primer (5′-CCCTTACCCTTACCCTTACCCTAA-3′) under a wax barrier (Ampli-wax; Perkin-Elmer, Foster City, CA, USA). Each tumour extract was assayed above the wax barrier in 50 μl of reaction mixture containing 5 μl of 10 × TRAP buffer (200 mM Tris-HCl pH 8.3, 15 mM magnesium chloride; 630 mM potassium chloride; 0.05% Tween 20; 10 mM EGTA; 1 mg $ml^{-1}$ bovine serum albumin), 1 μl of 2.5 mM dNTPs, 1 μl of TS primer (5′-AATCCGTCGAGCAGAGTT-3′; 0.1 μg $\mu l^{-1}$), 0.4 μl (2 U) *Taq* polymerase (Boehringer Mannheim), 40.2 μl of DEPC (diethyl pyrocarbonate) water, 0.4 μl of [α-$^{32}$P]dCTP (3000 Ci $mmol^{-1}$, Amersham) and 2 μl of telomerase extract (0.6–6.0 μg of protein). The tube was then incubated at room temperature for 20 min before polymerase chain reaction (PCR) amplification (25 cycles of 94°C/30 s, 50°C/30 s, 72°C/30 s). Four microlitres of loading dye containing bromophenol blue was then added. The products were run on 15% non-denaturing PAGE gel in 0.6 × TBE. The gel was then dried and the TRAP products were visualized by autoradiography. RNAase control was set up with each tumour extract by preincubating with 0.5 μg of DNAase-free RNAase at room temperature for 15 min.

© Cancer Research Campaign 1998

**Table 1** Telomerase activities in biopsied nasopharyngeal carcinomas

| Case no. | Sex | Age (years) | Clinical staging[a] | Ho's staging[b] | Telomerase activity | Presence of tumour cells | EBV serology (IgA to VCA titres) | Keratin |
|---|---|---|---|---|---|---|---|---|
| *Normal nasopharyngeal mucosa* | | | | | | | | |
| HKNP3 | M | 38 | | | – | No | | Positive |
| HKNP8 | M | 29 | | | – | No | | Positive |
| HKNP9 | F | 44 | | | – | No | | Positive |
| HKNP17 | F | 17 | | | – | No | | Positive |
| HKNP25 | M | 38 | | | – | No | | Positive |
| HKNP26 | M | 41 | | | – | No | | Positive |
| HKNP32 | M | 44 | | | – | No | | Positive |
| HKNP39 | F | 31 | | | – | No | | Positive |
| HKNP46 | F | 67 | | | – | No | | Positive |
| *Primary nasopharyngeal carcinomas* | | | | | | | | |
| HKNPC5 | M | 49 | I | T1N0 | – | Yes | 1:640 | Positive |
| HKNPC10 | F | 42 | I | T1N0 | + | Yes | 1:640 | Positive |
| HKNPC18 | F | 46 | I | T1N0 | + | Yes | < 1:10 (Negative) | Positive |
| HKNPC22 | M | 57 | I | T1N0 | + | Yes | 1:640 | Positive |
| HKNPC28 | M | 48 | I | T1N0 | + | Yes | 1:640 | Positive |
| HKNPC24 | F | 47 | II | T1N1 | + | Yes | 1:640 | Positive |
| HKNPC30 | F | 46 | II | T1NI | + | Yes | 1:640 | Positive |
| HKNPC4 | M | 37 | II | T2N0 | – | Yes | 1:160 | Positive |
| HKNPC20 | M | 53 | II | T2N0 | – | Yes | 1:640 | Positive |
| HKNPC54 | M | 60 | II | T2N0 | – | Yes | > 1:640 | Positive |
| HKNPC44 | M | 70 | II | T2N0 | + | Yes | < 1:10 (Negative) | Positive |
| HKNPC19 | M | 68 | II | T2N1 | + | Yes | 1:640 | Positive |
| HKNPC21 | F | 67 | II | T2N1 | + | Yes | 1:640 | Positive |
| HKNPC29 | F | 52 | II | T2N1 | + | Yes | 1:640 | Positive |
| HKNPC31 | M | 49 | II | T2N1 | + | Yes | 1:640 | Positive |
| HKNPC6 | M | 46 | III | T2N2 | + | Yes | 1:640 | Positive |
| HKNPC9 | M | 63 | III | T1N2 | + | Yes | 1:640 | Positive |
| HKNPC40 | F | 20 | III | T1N2 | + | Yes | 1:640 | Positive |
| HKNPC42 | M | 67 | III | T2N2 | + | Yes | 1:640 | Positive |
| HKNPC45 | M | 45 | III | T3N0 | + | Yes | 1:40 | Positive |
| HKNPC16 | M | 41 | IV | T2N3 | + | Yes | 1:640 | Positive |
| HKNPC23 | F | 37 | IV | T3N2 | + | Yes | 1:40 | Positive |
| HKNPC26 | M | 41 | IV | T3N3 | + | Yes | 1:640 | Positive |
| HKNPC27 | M | 73 | IV | T3N1 | + | Yes | 1:640 | Positive |
| HKNPC35 | M | 26 | IV | T3N1 | + | Yes | 1:160 | Positive |
| HKNPC39 | F | 31 | IV | T1N3 | + | Yes | 1:640 | Positive |
| HKNPC53 | M | 52 | IV | T1N3 | + | Yes | 1:20 | Positive |
| *Recurrent cancers (neck node metastasis)* | | | | | | | | |
| HKNPC7A | M | 34 | | | + | Yes | NA | Positive |
| HKNPC2 | M | 52 | | | + | Yes | 1:160 | Positive |
| HKNPC3 | M | 39 | | | + | Yes | 1:160 | Positive |
| HKNPC7 | M | 44 | | | + | Yes | 1:40 | Positive |
| HKNPC13 | M | 65 | | | + | Yes | 1:80 | Positive |
| HKNPC14 | M | 62 | | | + | Yes | 1:10 | Positive |
| HKNPC34 | F | 26 | | | + | Yes | NA | Positive |
| HKNPC49 | M | 62 | | | + | Yes | 1:640 | Positive |
| HKNPC51 | M | 65 | | | + | Yes | 1:80 | Positive |

[a]Wei and Sham (1994); [b]Wei (1984); EBV serology, diagnostic titres: 1:10; NA, not available.

### Western analysis of cytokeratin in NPC biopsies

Apart from histological confirmation of tumour cells in the NPC biopsies, we have also examined the presence of keratin in these biopsies to confirm the presence of epithelial cells (Tsao et al, 1995). The detection of keratin was particularly useful in establishing the presence of epithelial cells in normal nasopharyngeal biopsies, which were often small in size (1 $mm^3$). The cytokeratin extract was solubilized in loading buffer, resolved by 12% SDS-PAGE and transferred to PVDF membrane for immunodetection using AE1 and AE3 antibodies (Zymed Laboratories, South San Francisco, CA, USA) against cytokeratins.

### RESULTS

Telomerase activity was present in 32 out of 36 NPC specimens (89%) and in none out of nine non-neoplastic nasopharyngeal tissues (0%) (Figure 1).

The telomerase activities of all the 36 NPC biopsies are shown in Table 1. Positive telomerase activity was detected in 23 out of 27 biopsies (85%) obtained from primary NPC and in nine out of nine biopsies of recurrent NPC (100%). Telomerase activation was common in all stages of NPC: 80% in stage I (four out of five); 70% in stage II (seven out of ten); 100% in stage III (five out of five) and 100% in stage IV (seven out of seven). There was,

© Cancer Research Campaign 1998

**Table 2** Telomerase activity in established cell lines of nasopharyngeal carcinoma

| Nasopharyngeal carcinomas | Telomerase activity |
|---|---|
| *Xenografted tumours* | |
| 2117 | + |
| 666 | + |
| MET1 | + |
| MET2 | + |
| *In vitro cell lines* | |
| HK1 | + |
| 666 | + |
| CNE1 | + |
| CNE2 | + |
| 391 | + |
| *Normal nasopharyngeal epithelial cells* | |
| HKNP1 | – |

**Figure 2** Telomerase activity in established carcinoma cell lines. HK1, CNE1, CNE2 and 666 are established nasopharyngeal carcinoma cell lines. SiHa is an established cell line of cervical carcinoma. +, Telomerase assay conducted in the presence of RNAase inhibition (0.5 µg per reaction). –, Telomerase assay conducted in the absence of RNAase. Tissue extract was used at a protein concentration of 6 µg per reaction however, no statistical significance observed between biopsies from early stages (stage I and II) and advanced stages (stage III and IV) ($P > 0.05$; Fisher exact test) of NPC. However, telomerase activation in NPC biopsies with negative lymph node involvement appeared to be lower (six out of ten cases, 60%) than biopsies with positive lymph node involvement (17 out of 17 cases, 100%). The difference was statistically significant ($P < 0.05$, Fisher exact test). The status of positive and negative lymph node involvement was confirmed by computerized tomography (CT) and clinical examination. All the telomerase activities detected were sensitive to RNAase inactivation, which confirmed the specificity of the assay.

Activation of telomerase was common in established cell lines of NPC (Table 2). All established NPC cell lines examined were positive for telomerase activity. These included four xenografted NPC lines (2117, 666, MET1, MET2) (Figure 1) established in nude mice and four in vitro NPC cell lines (CNE1, CNE2, HK1, 391, 666) (Figure 2).

### DISCUSSION

This is the first report of telomerase activation in NPC. In line with observations in other human malignancies (Kim et al, 1994; Chadeneau et al, 1995), telomerase activation is also a common event in NPC. Telomerase activity can be detected at high frequency (89%) in NPC biopsies and in all NPC cell lines examined. Non-neoplastic nasopharyngeal mucosae were negative for telomerase activity. This is in agreement with the observation that telomerase activation is closely associated with malignancies but not with hyperplasia or benign tumours in other human cancers (Chadeneau et al, 1995; Tahara et al, 1995; Hiyama et al, 1996; Sommerfeld et al, 1996).

The association of telomerase with cell immortalization has been demonstrated in several cell systems, including EBV-immortalized human lymphocytes (Counter et al, 1994). It was shown that telomerase activation was not detected in the early phase of proliferation of B-lymphocytes after infection with EBV. Telomerase activity was detected only in the immortalized B-cell clones at the later stage of EBV immortalization. This unique property of telomerase distinguishes it from other proliferative markers, such as PCNA and Ki67, which are closely associated with proliferation. In this study, we only detected telomerase activation in NPC tissues but not in non-neoplastic nasopharyngeal biopsies. This is in line with the general observation that telomerase is detected in immortalized and cancer cells but not in mortal somatic cells. In NPC, telomerase activation was observed in all stages of diseases, suggesting that it is an early event in tumour progression. A higher frequency of telomerase activation was observed in advanced NPC compared with early diseases, however the difference is not statistically significant ($P > 0.5$). The sample size in this study may be small for the observation to be conclusive. There were four histologically confirmed NPC biopsies (all from early stages) with negative telomerase activity. The clinical significance of these telomerase-negative NPCs is not fully understood at this stage; the clinical history of these telomerase-negative NPC patients will be followed closely to examine any influence on survival or recurrence of the disease. Another observation is that telomerase activation appears to be more frequent in NPC biopsies with positive lymph node metastasis (100% positive rate) than those without lymph node involvement (60%) ($P < 0.05$, Fisher exact test), suggesting a positive association of telomerase activity with tumour progression. Other clinical features examined included tumour differentiation and serological EBV markers, but no obvious relationship with telomerase activation has been observed (Table 1).

In view of the high frequency of telomerase activation in NPC, the detection of telomerase activity alone may have limited prognostic value for NPC patients. The prognostic value of the level of telomerase activation in NPC remains to be determined. However, the high frequency of telomerase activation in NPC biopsies and its close association with tumour cells suggests that it may be useful as a malignant marker for the detection of tumour cells in NPC biopsies. Hence, the telomerase activity may complement the existing methods used for the diagnosis of NPC, such as serological markers (e.g. IgA/EBV, IgA/EA) and the PCR method for EBV. In support of this, we were able to detect telomerase activity in two NPC biopsies (HKNPC 18 and 44) that were seronegative for EBV VCA. As EBV is present in both NPC cells and lymphocytes in biopsies, the use of the PCR method alone will not give conclusive evidence for the presence of tumour cells in NPC biopsies. The telomerase assay has the advantage over the PCR method in detecting not only EBV-positive cells but also immortalized tumour cells. The detection of high telomerase activity in nasopharyngeal biopsies may warrant further examinations, including

© Cancer Research Campaign 1998

repeated biopsies from the patients and careful monitoring of serological markers for NPC. Another potential application of the telomerase assay may be in the detection of submicroscopic tumour foci or premalignant but immortal lesions that may escape histopathological examination.

### ACKNOWLEDGEMENTS

The authors would like to express their thanks to Dr CP Chiu, Geron Company, Cupertino, CA, USA, for her advice in the establishment of the TRAP assay. This project is supported by a grant (HKU 7325/97M) from the Research Grant Committee, Hong Kong, and a CRCG grant (337/031/0037) from the University of Hong Kong.

### REFERENCES

Avilion AA, Piatyszek MA, Gupta J, Shay JW, Bacchetti S and Greider CW (1996) Human telomerase RNA and telomerase activity in immortal cell lines and tumor tissues. *Cancer Res* **56**: 645–650

Chadeneau C, Hay K, Hirte HW, Gallinger S and Bacchetti S (1995) Telomerase activity associated with acquisition of malignancy in human colorectal cancer. *Cancer Res* **55**: 2533–2536

Counter CM, Botelha FM, Wang P, Harley CB and Bacchetti S (1994) Stabilization of short telomeres and telomerase activity accompany immortalization of Epstein-Barr virus-transformed human B lymphocytes. *J Virol* **68**: 3410–3414

Gu SY, Tang WP, Zeng Y, Zhao ML, Zhao EWP, Deng WH and Li Ki (1983) An epithelial cell line established from poorly differentiated nasopharyngeal carcinoma (in Chinese) *Chinese J Cancer* **2**: 70–72

Hiyama E, Hiyama K, Yokoyama T, Matsuura Y, Piatyszek MA and Shay JW (1995*a*) Correlating telomerase activity levels with human neuroblastoma outcomes. *Nature Med* **1**: 249–257

Hiyama E, Yokoyama T, Tatsumota N, Hiyama K, Imamura Y, Murakami Y, Kodama T, Piatyszek MA, Shay JW and Matsuura Y (1995*b*) Telomerase activity in gastric cancer. *Cancer Res* **55**: 3258–3262

Hiyama E, Gollahon L, Katoka T, Katsumasa K, Yokoyama T, Gazdar AF, Hiyama K, Piatyszek MA and Shay JW (1996) Telomerase activity in human breast tumors. *J Natl Cancer Inst* **88**: 116–122

Huang DP (1991) Epidemiology and etiology of nasopharyngeal carcinoma. In *Nasopharyngeal Carcinoma*, van Hasselt CA and Gibb AG. (eds), pp. 23–35, Chinese University Press: Hong Kong

Huang DP, Ho JHC, Poon YG, Chew EC, Saw D, Liu M, Li CL, Mak LS, Lai SH and Lau EH (1980) Establishment of a cell line (NPC/HK1) from a differentiated squamous carcinomas of the nasopharynx. *Int J Cancer* **26**: 127–132

Huang DP, Ho JHC, Chan WK, Lau WH and Lui M (1989) Cytogenetics of undifferentiated nasopharyngeal carcinoma xenografts from southern Chinese. *Int J Cancer* **43**: 926–939

Huang DP, Lo KW, Choi PHK, Ng Angela YT, Tsao SY, Yiu GKC and Lee JCK (1991) Loss of heterozygosity on the short arm of chromosome 3 in nasopharyngeal carcinoma. *Cancer Genet Cytogenet* **54**: 91–99

Huang DP, Lo KW, van Hasselt A, Woo JKS, Choi PHK, Leung SF, Cheung ST, Cairns P, Sidransky D and Lee JCK (1994) A region of homozygous deletion on chromosome 9p21–22 in primary nasopharyngeal carcinoma. *Cancer Res* **54**: 4003–4006

Kastury K, Baffa R, Druck T, Ohta M, Cotticelli MG, Inoue H, Negrini M, Rugge M, Huang D, Croce CM, Palazzo J and Huebner K (1996) Potential gastrointestinal tumor suppressor locus at the 3p14.2 FRA3B site identified by homozygous deletions in tumor cell lines. *Cancer Res* **56**: 978–983

Kim NW, Piatyszek MA, Prowse KR, Harley CB, West MD, Ho PC, Coviella GM, Wright WE, Weinrich SL and Shay JW (1994) Specific association of human telomerase activity with immortal cells and cancer. *Science* **266**: 2011–2015

Lo KW, Tsao SW, Leung SF, Choi PHK, Lee JCK and Huang DP (1994) Detailed deletion mapping on the short arm of chromosome 3 in nasopharyngeal carcinomas. *Int J Oncol* **4**: 1359–1364

Lo KW, Huang DP and Lau KM (1995) p16 gene alterations in nasopharyngeal carcinoma. *Cancer Res* **55**: 2039–2043

Muir C, Warehouse J, Mack T, Powell J and Whelan S (1987) *Cancer Incidence in Five Continents*, Vol. 5. IARC Scientific Publication No. 88. International Agency for Research on Cancer: Lyon, France

Ohta M, Inouse H, Cotticelli MG, Kastury RB, Palazzo J, Siprashvili Z, Mori M, McCue P, Druck T, Carce CM and Huebner K (1996) The FHIT gene, spanning the chromosome 3p14.2 fragile site and renal carcinoma-associated t(3;8) breakpoint, is abnormal in digestive tract cancers. *Cell* **84**: 587–597

Piatyszek MA, Kim NW, Weinrich SL, Hiyama K, Hiyama E, Wright WE and Shay JW (1995) Detection of telomerase activity in human cells and tumors by a telomeric repeat amplification protocol (TRAP). *Method Cell Sci* **17**: 1–15

Spruck CH III, Tsao YC, Huang DP, Yang AS, Rideout WM III, Gonzalwz-Zulueta M, Choi P, Lo KW, Yu MC and Jones PA (1992) Absence of p53 gene mutations in primary nasopharyngeal carcinomas. *Cancer Res* **52**: 4787–4790

Sun Y, Hegamyer G and Colburn NH (1993) Nasopharyngeal carcinoma shows not detectable retinoblastoma susceptibility gene alterations. *Oncogene* **8**: 791–795

Tahara H, Nakanishi T, Kitamota M, Nakashio R, Shay JW, Tahara E, Kajiyama K and Toshinori (1995) Telomerase activity in human liver tissues: comparison between chronic liver disease and hepatocellular carcinomas. *Cancer Res* **55**: 2734–2736

Tsao SY (1991) Radiotherapy of nasopharyngeal carcinoma. In *Nasopharyngeal Carcinoma*. van Hasselt CA and Gibb AG. (eds), pp. 189–211. Chinese University Press: Hong Kong

Tsao SW, Mok SC, Fey EG, Fletcher JA, Wan TS, Chew EC, Muto MG, Knapp RC and Berkowitz RS (1995) Characterization of human ovarian surface epithelial cells immortalized by human papilloma viral oncogenes (HPV-E6E7 ORFs). *Exp Cell Res* **218**: 499–507

Sommerfeld H-J, Meeker AK, Piatyszek MA, Bova GS, Shay JW and Coffey DS (1996) Telomerase activity: a prevalent marker of malignant human prostate tissue. *Cancer Res* **56**: 218–222

Wei WI (1984) A comparison of clinical staging systems in nasopharyngeal carcinoma. *Clin Oncol* **10**: 225–231

Wei WI and Sham JST (1994) Nasopharyngeal Carcinoma. In *Oxford Textbook of Surgery*, Morris PJ and Malt RC. (eds), pp. 2540–2544. Oxford University Press: New York

© Cancer Research Campaign 1998